# The expression analysis of *Fra-1* gene and IL-11 protein in Iranian patients with ulcerative colitis

**DOI:** 10.1186/s12865-018-0257-9

**Published:** 2018-06-18

**Authors:** Milad Sabzevary-Ghahfarokhi, Mojtaba Shohan, Hedayatollah Shirzad, Ghorbanali Rahimian, Nader Bagheri, Amin Soltani, Fatemeh Deris, Mahdi Ghatreh-Samani, Ehsan Razmara

**Affiliations:** 1 Department of Microbiology and Immunology, Faculty of Medicine, Shahrekord University of Medical Sciences, Shahrekord, Iran; 2 Cellular and Molecular Research Center, Basic Health Sciences Institute, Shahrekord University of Medical Sciences, Shahrekord, Iran; 3 Department of Internal Medicine, Shahrekord University of Medical Sciences, Shahrekord, Iran; 4 Medical Plants Research Center, Basic Health Sciences Institute, Shahrekord University of Medical Sciences, Shahrekord, Iran; 5 Department of Epidemiology and Biostatistics, School of Health, Shahrekord University of Medical Sciences, Shahrekord, Iran; 6 Department of Medical Genetics, Faculty of Medical Sciences, Tarbiat Modares University, Tehran, Iran

**Keywords:** Oxidative stress, Ulcerative colitis (UC), *Fra-1* gene, IL-11

## Abstract

**Background:**

Fra-1 (fosl1) belongs to the activator protein1 (AP-1) family inducing *IL-11* expression in oxidative stress condition. IL-11 plays a pivotal role in protecting epithelial barriers integrity. In this study, we investigated the *Fra-1* gene expression in the inflamed mucosa of patients with ulcerative colitis (UC) as well as its relation to IL-11 expression.

**Materials and methods:**

We enrolled 20 patients and 20 healthy controls with definite UC based on the clinical criteria. *Fra-1* gene expression in inflamed and non-inflamed colonic biopsies was determined by real-time polymerase chain reaction (RT-PCR). The IL-11 protein concentration was measured by Enzyme-Linked Immunosorbent Assay (ELISA) method. Pearson correlation was applied to calculate the relation between Fra-1 and IL-11.

**Results:**

An increased level of *Fra-1* gene expression was observed in patients with mild ulcerative colitis. The protein concentration of IL-11 was also increased in mild UC patients. Conversely, a significant decrease of IL-11 protein level was detected in severe UC patients compared to control group.

**Conclusion:**

Oxidative stress in inflamed intestinal biopsies can induce *fra*-1 gene expression. Our findings suggest that Fra-1 transcription factor leads to the production of IL-11 protein in UC patients.

## Background

The inflammatory bowel diseases (IBDs) consist of a wide range of disorders, like ulcerative colitis (UC), beginning in early adulthood and affecting the remaining life-span [1, 2]. UC is pathologically diagnosed by inflammation and injury in the gastrointestinal tract. Various factors are involved in its pathogenesis [3]. Despite extensive investigations on patients and experimental models, the main causes of UC, in terms of aetiology have not been understood [4]. Recent investigations have reported that oxidative stress plays crucial roles in the pathogenesis of UC [5]. The release of reactive oxygen species (ROS) accompanied by up-regulation of proinflammatory cytokines in intestinal inflammation which leads to activation of various intracellular pathways, such as mitogen-activated protein kinase (MAPK), signal transducer and activator of transcription 3 (STAT3) [6–8]. The activator protein1 (AP-1) family is an intracellular factor modulated by MAPK pathway. AP-1 is a homodimer or heterodimer of Fos (c-Fos, Fos B, Fra-1, and Fra-2), Jun (c-Jun, Jun D, and Jun B), and activating transcription factor (ATF-1 and ATF-2) proteins [9].

**Fig. 1:**
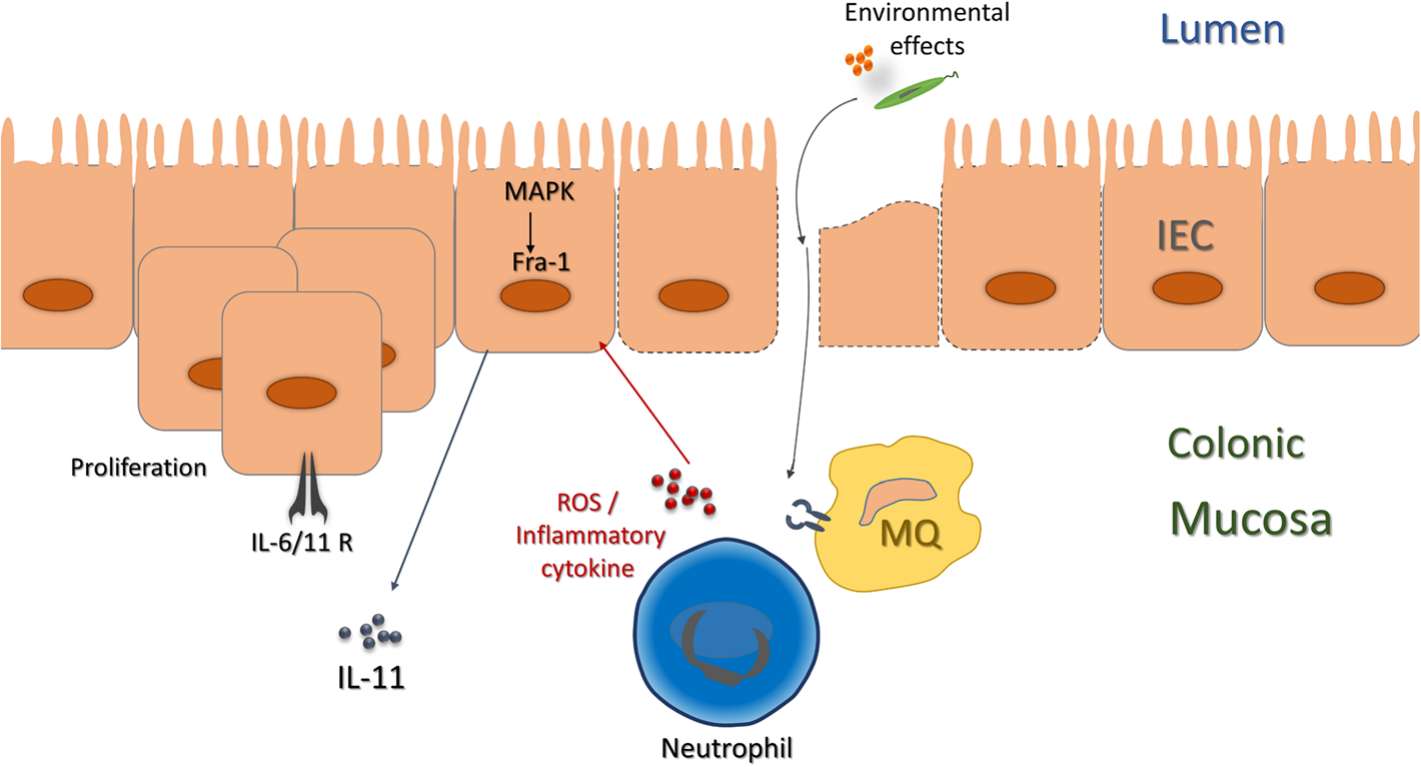
The IL-11/fra-1 pathway that in colonic tissue of patients with UC is designed. Environmental effects trigger innate immune system to produce ROS and inflammatory mediators. IL-11 can be released to reconstruct injury as a result of the activated IL-11/fra-1 pathway

Fra-1 (fosl1) is one of the main AP-1 family transcription factors with diverse functions particularly in epithelial cell growth, differentiation, and transformation. Fra-1 contributes to creating Epithelial-to-mesenchymal transition (EMT) and further carcinogenesis [9, 10]. an inflammatory cytokine, like interleukin-6 (IL-6), potentially triggers *Fra-1* gene transcription by binding STAT3 to the promoter of *Fra-1* gene in colorectal carcinoma (CRC) cells [11]. In mice model, blocking of AP-1 transcription factor has been shown to inhibit colonic inflammation during DSS-induced colitis [12]. On a cellular level, oxidative stress has been shown to induce IL-11 production via Fra-1 signalling [13] (Fig. 1). Likewise, Fra-1 along with Nuclear Factor Erythroid 2-related Factor 2 (NRF2), motivates IL-11 production in extra electrophiles environments [14].

IL-11, a member of the IL-6 family, is strongly expressed in an oxidative stress condition [15]. IL-11 binds to IL-11 receptor alpha (IL-11Rα) on various cells including lung and intestinal epithelial cells as well as osteoclast cell surfaces. IL-11 are potentially capable of hematopoiesis, repair mechanisms, bone development and carcinogenesis [6, 15]. IL-11 and IL-6 activate STAT3 through GP130 signalling so that intestinal cells are highly regenerated after damage [6]. IL-11 may be involved in inflammatory responses and development of colitis-associated colorectal cancer [16]. On the other hand, IL-11 can improve intact intestinal barrier in mice model colitis by up-regulation of TLR-2 in the colon [17].

In this study, we will examine the IL-11 protein amount in the inflamed colonic tissue of the patients affected by ulcerative colitis. However, this analysis was not particularly applied in patient’s epithelial cells. Besides, *Fra-1* gene expression and its relationship with IL-11 were considered in the following. In this paper, we demonstrate the overexpression of Fra-1 gene is due to its transcription factor as observed in mild UC patients and suggest that IL-11 is elevated in inflamed colonic tissues.

## Materials and methods

### Sample collection and preparation

The study group comprised of 20 patients with UC and 20 healthy individuals as control (Table.1). All cases were attended in Gastroenterology Unit of Hajar hospital, Shahrekord, Iran, from January to May 2017. Clinical information of the patients was collected based on an opt query and medical files consisting of age, gender, medical and surgical history, food habits, stress, disease severity and extension, colonoscopic and pathologic records, treatment and hospitalizations. Disease activity index for ulcerative colitis was evaluated by the combination of endoscopic and clinical scale [18]. The diagnosis of UC was confirmed by the related clinical manifestations such as abdominal pain, chronic diarrhea, anal bleeding, and histological criteria according to the Montreal Classification [19]. Fourteen UC patients had a mild proctosigmoiditis (inflammation was limited from the colorectum distal to the splenic flexure/ erythema is detected in the rectum and sigmoid, decreased friability and vascular pattern/ Montreal class: E2). Six severe patients had consumed corticosteroid six months prior the sampling date (Involvement extends proximal to the splenic flexure/ Montreal class: E3). The control group was selected among healthy individuals without any immune-mediated diseases such as multiple sclerosis and arthritis rheumatoid. These subjects were undergone colonoscopy due to the screening for colorectal cancer or polyp surveillance without inflammatory disease or any medication. The study was approved by Shahrekord University of Medical Sciences Ethics Committee. The written consent was also obtained from all individuals in the study.

**Table 1:** Demographic information of the patients

	Control	Mild colitis	Severe colitis	*P*-value
Age	33.81 ± 8.85	35.43 ± 9.55	32.00 ± 9.06	0.729
Gender (M/F)	11/9	7/7	3/3	0.913
Smoking %	9.5%	21.4%	46.7%	0.019*
Habitat (Rural/Urban)	2/19	4/10	3/3	0.078
Previous Abdominal Surgery	19%	64.3%	50%	0.295
Montreal Classification of Extent (E index)		E1: 3 E2: 11	E3: 6	0.674
Montreal Classification of Severity (S index)		S1: 8 S2:4	S3: 6	0.756

*= The number of smoking people were reach statistical significance (*P*- value = 0.019)

*E1* ulcerative proctitis, *E2* left-sided UC (distal to splenic flexure), *E3*: extensive (proximal to splenic flexure)

*S0* clinical remission, *S1*: mild UC, *S2* moderate UC, *S3*: severe UC

### Enzyme-linked immunosorbent assay

Two samples or colonic biopsies were taken from each individual by a GI specialist. The biopsies were kept at − 80 °C and the *Fra-1* gene and IL-11 protein were detected afterward. The samples’ protein were extracted using an Abcam ELISA lysis buffer guide [20].

Briefly, we prepared extraction buffer by (100 mM Tris, 150 mM NaCl, 1 mM EDTA, 1% Triton X-100 and 0.5% Sodium deoxycholate) and 1 mL of extraction buffer pipetted to each tube. It was followed by adding PMSF and Protease inhibitor cocktail to the tube and homogenized one biopsy in each tube. The supernatant of each sample was separated after 20 min centrifugation at 13000 rpm at 4 °C. Total protein concentrations were measured by Bradford protein assay and normalized individually. All samples were evaluated in two replicates. The limit of detection was 10 pg/mL. and of those > 10 pg/mL as considered as positive. Quantification of Human IL-11 protein Level was measured by means of Abcam ELISA kit for human IL-11 (Abcam, England, catalog number: ab100551), according to the manufacturer instructions.

### RNA isolation and quantitative RT-PCR

Total RNA was extracted from biopsies using TRIzol® reagent (Invitrogen/Thermo Fisher Scientific, Inc., catalogue number. 15596026) For each sample, RNA concentration was determined by Thermo Scientific™ NanoDrop 2000 and stored at − 80 °C. For all samples, 260/280 ratio for samples was greater than 1.8. cDNAs were synthesized using the RevertAid first-strand cDNA synthesis kit (Thermo Scientific, K1622) with 1.5 μg of RNA in a reaction volume of 20 μL after DNaseI (Fermentas EN0521) digestion. The quantification of mRNA was performed using RT-PCR on a Rotor-Gene RG-300 (Corbett Research, Sydney, AU) and the SYBR Green Real-time PCR Master Mix Kit (TAKARA, Japan, catalog number. RR820Q) were used according to the protocol provided by the manufacturer. The primers were designed by Primer3.0 (http://bioinfo.ut.ee/primer3-0.4.0) web-based server (Table.2). We ensured that there were no Single Nucleotide Polyphemus in the genomic region corresponding to the 3′ ends of primers by looking through the dbSNP database. This was done to evaluate the ability of generating unique primers for our experiment. The primers specificity was confirmed and verified by the in-silico-PCR tool in UCSC genome browser and Primer blast of NCBI genome browser. Thermal cycling was initiated with a first denaturation step at 95 C for a duration of 5 min and was followed by 38 cycles of 95 °C for 15 s, 61 °C for 20s and 72 °C for 25 s. Melting curve analysis was used to confirm amplification specificities. Gene expression was normalized to internal controls and fold changes were calculated using relative quantification (2^−ΔΔCq^).

**Table 2:** Primer sequences used for real time PCR quantifications

Gene	Primer sequences (5′-3′)	Tm (°C)	Amplicon Size (bp)
*fra-1*	F: -5́ TGACCACACCCTCCCTAACTC -3́	60.83	100 bp
	R: -5́ CTGCTGCTACTCTTGCGATGA -3́	60.47	
GAPDH	F: -5́ ACAGTCAGCCGCATCTTC -3́	57.39	169 bp
	R: -5́ CTCCGACCTTCACCTTCC -3́	57.66	

### Statistical analysis

All data were presented as mean ± SD and were evaluated by SPSS19.0 (SPSS Inc., Chicago, IL, USA) and GraphPad Prism software version 5.0 (GraphPad Software, La Jolla, CA, USA). The distribution of data was normal and the relationship between age of subjects and groups was addressed with the Fischer exact test. Unpaired t-Test was used to compare the two groups and multiple comparisons were done by Tukey post hoc. Pearson’s correlation analysis was used to estimate the correlations between *Fra-1* gene expression and IL-11 protein amount. Differences calculating in *P*-values < 0.05 were considered to be statistically significant.

## Results

### *Fra-1* gene expression increased in mild colitis

**Fig. 2:**
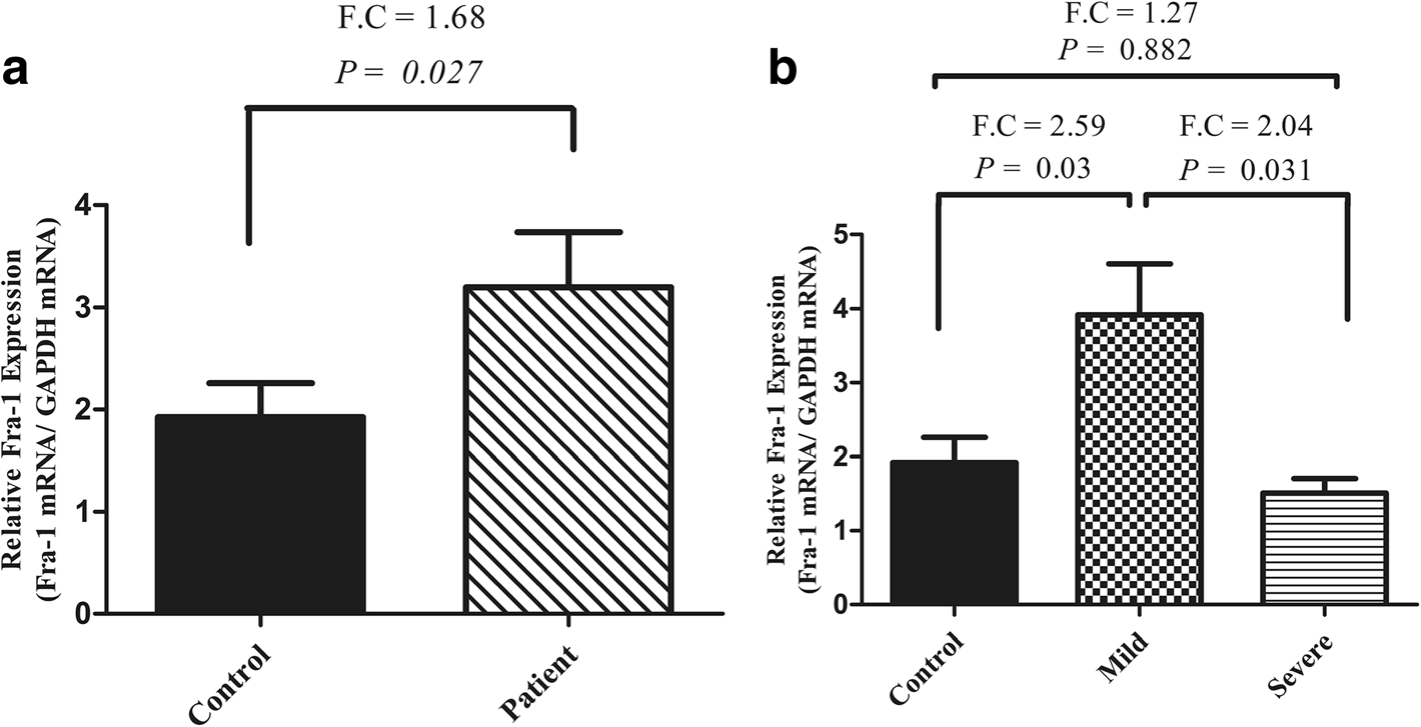
Fra-1 (fosl1) mRNA level was evaluated in 20 UC and 20 control mucosa**.** The data derived from the real-time PCR for human Fra-1 were normalized versus GAPDH-as an inner gene control. **a** Increased level of *Fra-1* mRNA was detected in the patient group (*P*-value = 0.027). **b** Similarly, *Fra-1* gene expression was dramatically overexpressed in endoscopic specimens in mild patients rather than in both severe patients and control. UC: ulcerative colitis. F.C: Fold Change

We evaluated the mRNA level of Fra-1 by RT-PCR in the inflamed colonic biopsy of mild and severe UC patients. In this experiment, *Fra-1* gene expression in UC patients was compared with control samples (taken from non-inflamed colonic mucosa). As shown in the Fig. 2a, the mean *Fra*-1 gene expression in UC samples seem to be more than control samples and the explanatory variables were statistically significant (*P*-value = 0.027). The gene expression of *Fra-1* in mild UC patients is twofold higher than the control group (*P*-value = 0.010, Fig. 2b). Moreover, the Fra-1 mRNA level was remarkably elevated in mild patients compared to that of severe (*P*-value = 0.031, Fig. 2b). Surprisingly, the comparison of *Fra-1* gene expression between severe and control biopsies did not show any statistical signification (*P*-value > 0.05, Fig. 2b).

### Expression of IL-11 protein is declined in severe colitis

**Fig. 3:**
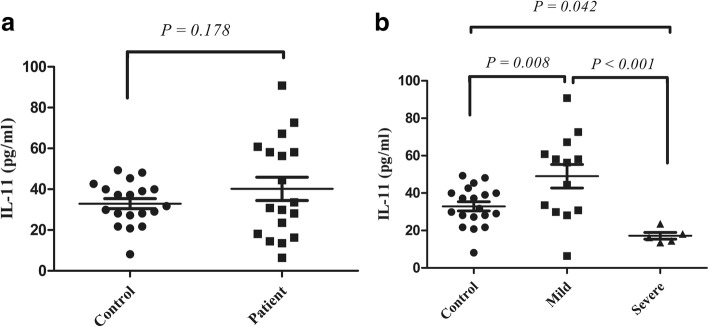
IL-11 protein amount was determined by ELISA. **a** IL-11 protein in patients showed the highest level; however, it is not statically significant (*P*-value > 0.05). **b** IL-11 protein in endoscopic specimens was elevated in mild patients compared to both severe patients and controls

Recent investigations have suggested that *IL-11* gene expression decreased with an extended intensity of inflammation [21], but our data somehow conflicting. IL-11 protein level was measured by ELISA method in colonic biopsies of UC patients. IL-11 protein concentrations in severe patients were dramatically declined (mean ± SD = 17.181 ± 3.96 pg/ml). On the other hand, the concentration for mild patients and controls had a much wider increase, at about 52.56 ± 19.62 and 34.28 ± 9.04 pg/ml, respectively (Fig. 3b). Accordingly, statistical analysis showed meaningful differences between mild UC and control’s group (*P*-value = 0.008), severe UC and control (*P*-value = 0.042), and mild UC and severe UC (*P*-value < 0.001) (Fig. 3b). On the other hand, no differences were found in IL-11 protein expression between UC patients and controls (*P*-value > 0.05, Fig. 3a). Furthermore, the protein levels of IL-11 considerably correlated with *Fra-1* gene expression in patients. This analysis indicates that increased *Fra-1* gene expression results in surging IL-11 in UC colonic biopsies (*r* = 0.704, Table 3).

**Table 3:** Pearson correlations for *Fra-1* gene expression and IL-11 protein concentration

Groups	Correlation coefficient (IL-11 protein*/ Fra-1* gene)	*(P-*value*)*
Control (*n* = 20)	−0.319	0.196
All Patient (20)	0.704	0.002*
Mild Patient (*n* = 14)	0.560	0.058
Severe Patient (*n* = 6)	0.021	0.973

*IL-11 protein correlated with *Fra-1* gene expression in patients and controls

## Discussion

The destructive effects of ROS are highly connected to cytokine responses involved in repairing intestinal injury when the oxidative damage in the inflamed mucosa correlates with IBD intensity [22]. The existence of specific polymorphism, dinucleotide repeat of the IL11.A1 allele, in IL-11 was significantly associated with UC have been seen in various studies [23]. Making use of recombinant human IL-11 able to maintain remission phase in Crohn’s disease [24]. Nevertheless, there are few different theories concerning the role that IL-11 plays to promote colitis into colorectal cancer while the alleviating function of IL-11 in colitis has been detected [16]. The direct effects of IL-11 can differentiate CD4^+^ T cells into Th2 cells which is a dominant subset of T effectors in UC [25]. In other Th2-dominant inflammatory diseases, like asthma, the high level of IL-11 has been measured [26]. A previous study reported the decreasing of *IL-11* gene expression in mild and severe UC patients, but did not assess IL-11 intestinal or serum protein levels [21]. In contrast to this study, we detected an enhanced IL-11 protein expression in mild UC. Some investigations on mice showed that the protective and restorative roles of IL-11 elevated when the murine intestinal cells were exposed to radiation or chemical stresses. IL-11 was considered as a mucosal protective in addition to retraining the apoptosis of mature enterocytes [27, 28]. Our data illustrated that *Fra-1* gene expression had a similar pattern with an expression of the IL-11 protein. Furthermore, *Fra-1* gene expression in our analysis positively correlated with IL-11 expression in the mild patient group. Fra-1 factor is an essential mediator to induce IL-11 through oncogenic Ras activation. The human pancreatic carcinoma cells decreased serum induce-IL-11 in response to Fra-1 blocking siRNA even after making use of Ras activator [29].Both Fra-1 and IL-11 are relatively overexpressed in oxidative stress condition. Extracellular signal-regulated kinase2 (ERK2), which is stimulated by ROS components, induces Fra-1 in retinal, liver and CRC cell [10, 13, 30]. Pro-inflammatory cytokines such as IL-18 and IL-6 are able to stimulate MAPK pathway leading to increasing epithelium-derived IL-11 in DSS mice model [11, 31]. It has been elucidated that the targeting AP-1 factor through oligodeoxynucleotide (ODN) therapy reduces histological inflammation in DSS-induced experimental murine colitis [12]. Oxidative stress, along with Fra-1 factor, can activate other transcription factors including NRF-2 and HIF-1 that up-regulate IL-11 transcription [32]. Mothers against decapentaplegic homolog 3 (SMAD3) is affected by microenvironment in UC patients and is altered into pSmad3L/C form. Accordingly, SMAD3 and Runt-related transcription factor 2 (RUNX2) bind to the *IL-11* promoter in order to transcript *IL-11* gene [33, 34]. These factors lead to the production of IL-11 protein in mild UC. However, IL-11 and *Fra-1* expression are decreased in severe UC mucosa remains unclear. Our observations suggest the possibility that consuming corticosteroid drug in severe UC patients may inhibit Fra-1 and IL-11 in the following. In fact the loss of AP-1 and NF-kB factors as a result of corticosteroids effects consequently inhibits IL-11 production [35, 36]. On the other hand, transforming Growth Factor beta (TGF-β1) which is an essential co-factors with Fra-1 to induce IL-11, is significantly decreased in active form of UC patients [37]. Otherwise, further investigations are needed to characterize molecular mechanisms responsible for IL-11 and *Fra-1* gene expression. In addition, specific cells that produce IL-11 in UC intestine should be clearly detected. In sum, we demonstrated that IL-11 protein expression is increased in the colonic biopsy of mild UC patients. Our data suggest that epithelial cells under oxidative stress trigger *Fra-1* and IL-11 expression in the following.

## Conclusion

The present findings demonstrate that *Fra-1* gene expression and protein levels of IL-11 could be influenced by ROS in intestine epithelial cells. In conclusion, *Fra-1* gene expression and IL-11 protein amounts are increased in the colonic biopsy of mild UC patients. This seems to be a repairing and protective mechanism against injury in UC patients; however, more reliable research will be carried out to shed light on the reasons why IL-11 is declined in severe UC.

## Abbreviations

AP-1: Activator protein1
CRC: Colorectal carcinoma
EMT: Epithelial-to-mesenchymal transition
IBD: Inflammatory bowel diseases
IL-6: Interleukin-6
MAPK: Mitogen-activated protein kinase
NRF2: Nuclear Factor Erythroid 2-related Factor2
ROS: Reactive oxygen species
RUNX2: Runt-related transcription factor 2
SMAD3: Mothers against decapentaplegic homolog 3
STAT3: Signal transducer and activator of transcription 3
TGF-β1: Transforming Growth Factor beta 1
UC: Ulcerative colitis
